# Supplementary material for: Synthesis of polymers with on-demand sequence structures via dually switchable and interconvertible polymerizations
Source: Nat Commun. 2018 Jul 3;9:2577. doi: 10.1038/s41467-018-05000-2 (PMC6030099; doi:10.1038/s41467-018-05000-2)
Supplement: Supplementary file 1 — Supplementary Information [file 41467_2018_5000_MOESM1_ESM.pdf]

Zhang et al.

Synthesis of Polymers with On-Demand Sequence Structures via Dually Switchable and Controlled Interconvertible Polymerizations

Supplementary Figures

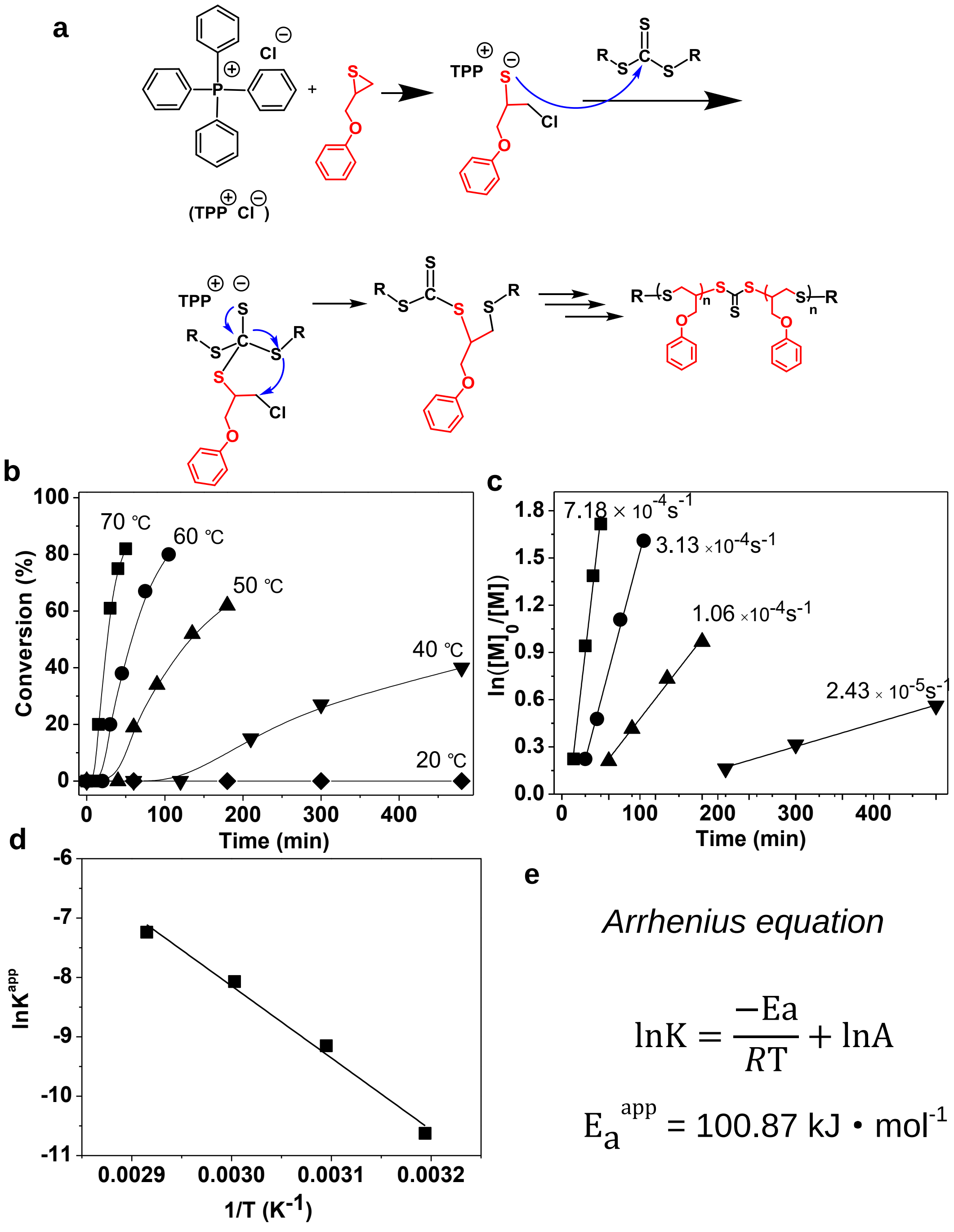

Supplementary Figure 1 AROP of thiirane monomer at different temperatures: a) Polymerization mechanism. b) Monomer conversion versus time. c) The variation of $\ln([M]_0/[M])$ with time. d) Relationship of apparent rate constant (K^{app}) with temperature. e) Apparent activation energy (E) calculated using Arrhenius equation.

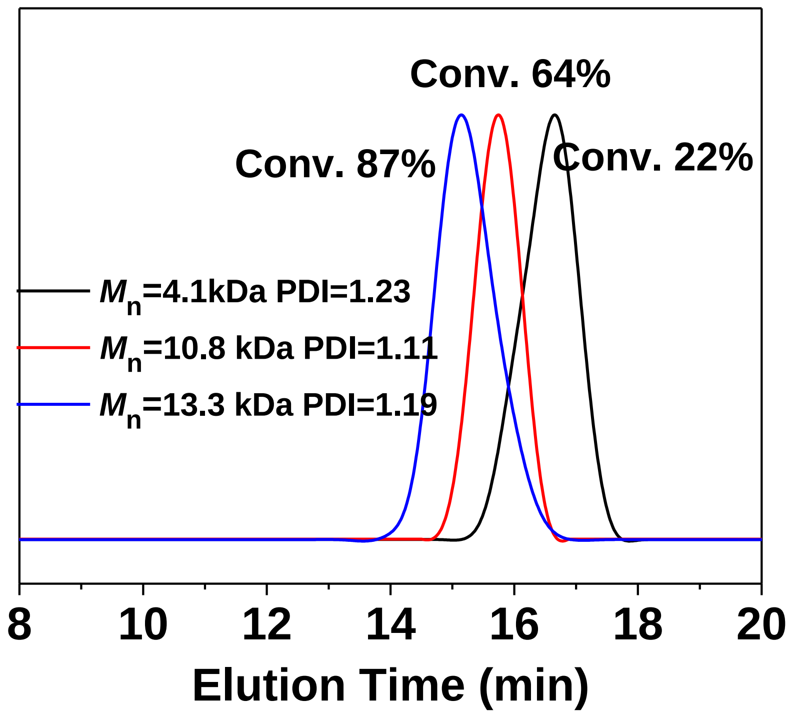

Supplementary Figure 2 SEC results of the polymers obtained at each stage during the switch ON/OFF experiment of AROP of POMT.

a

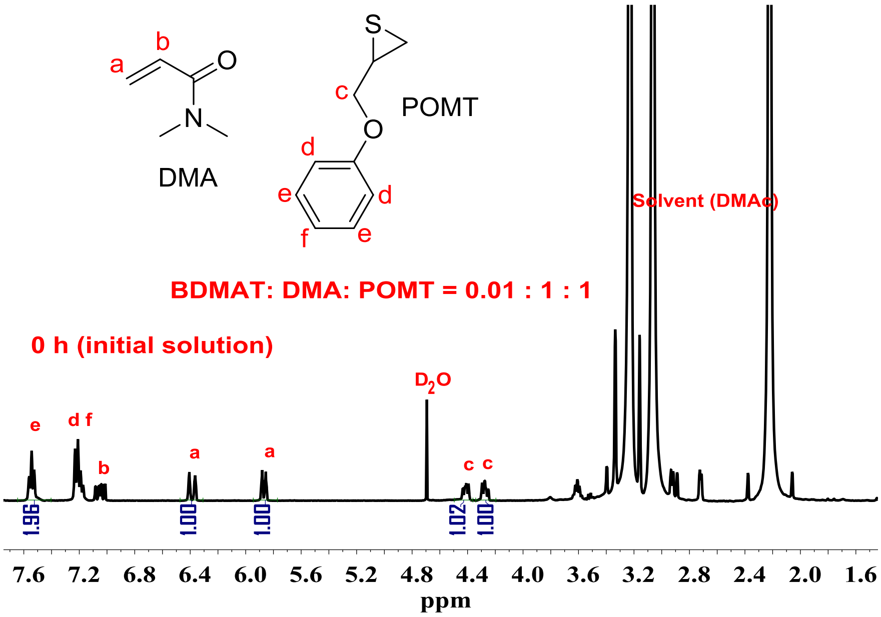

b

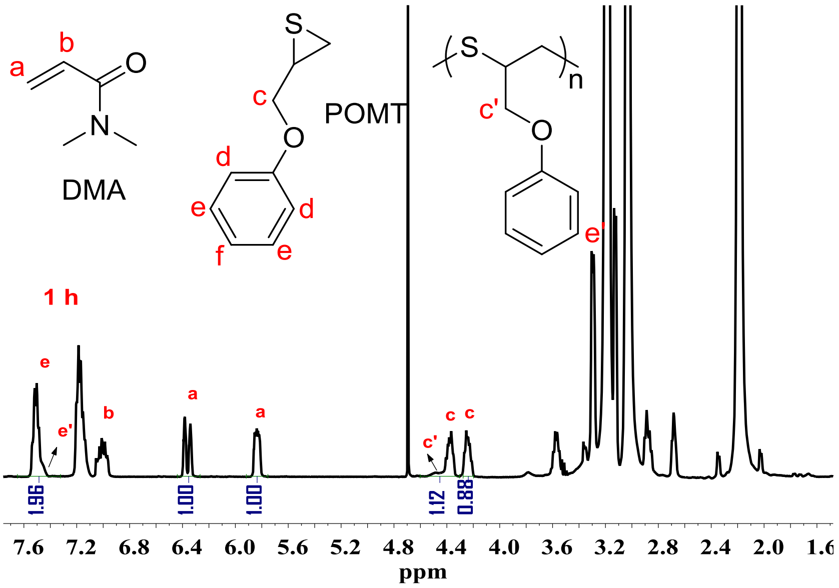

Supplementary Figure 3 ^{1}H–NMR trace showing the ON/OFF switch during the formation of ABA copolymer: a) Initial solution. b) 1 h after polymerization (1 h heating +0 h irradiation; conversion of POMT = 12%, conversion of DMA = 0%). Conversion (POMT) = $(1\text{-}I_1) \times 100\%$, Conversion (DMA) = $(1\text{-}I_2) \times 100\%$; I_1 denotes the protons integral values of methylene at unreacted POMT (δ = 4.24 ppm). I_2 denotes the protons integral values of methylene at unreacted DMA (δ = 5.88 ppm).

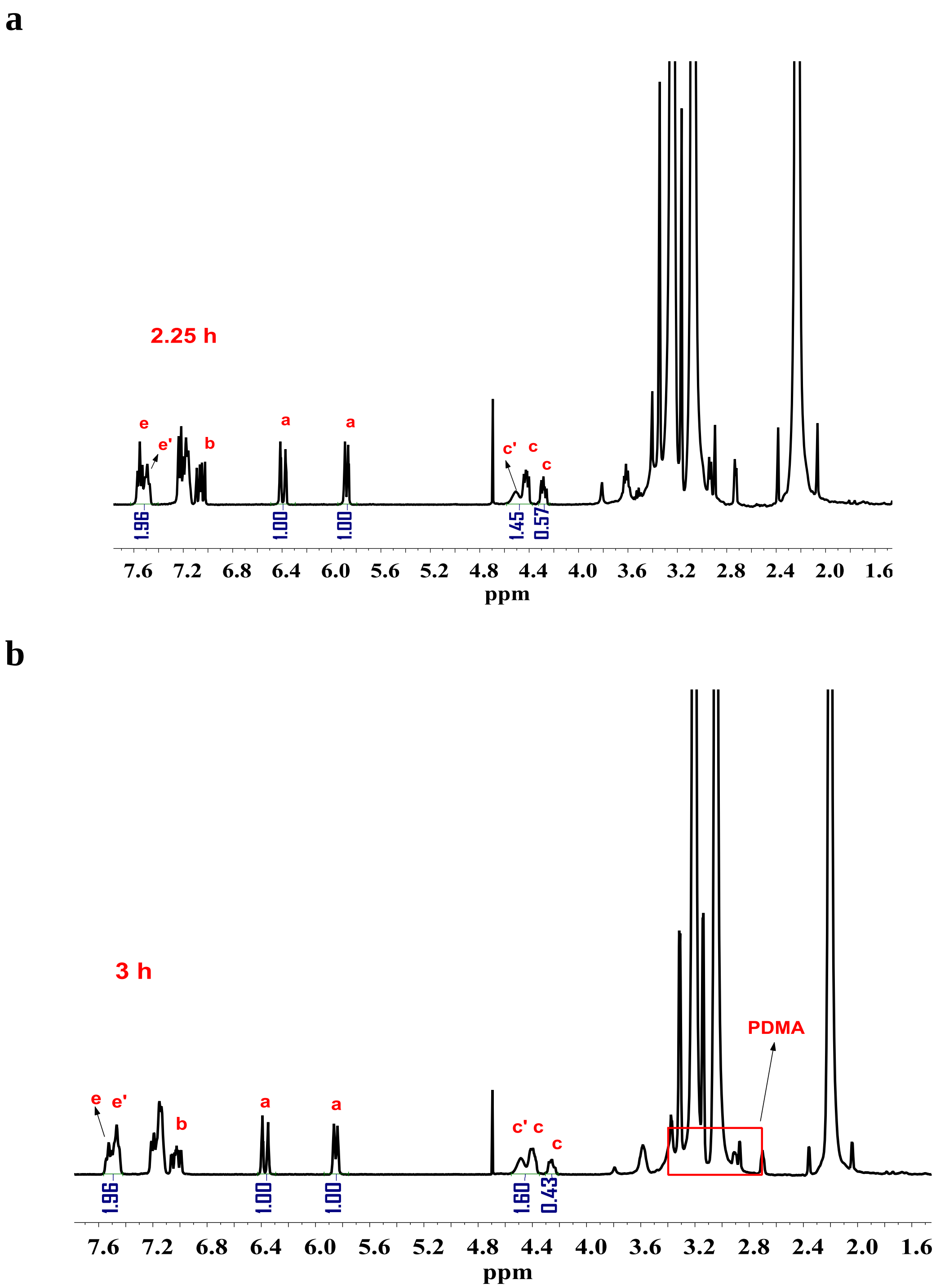

Supplementary Figure 4 ^{1}H–NMR trace showing the ON/OFF switch during the formation of ABA copolymer: a) 2.25 h after polymerization (2.25 h heating +0 h irradiation; conversion of POMT = 43%, conversion of DMA = 0%). b) 3 h after polymerization (3 h heating +0 h irradiation; conversion of POMT = 57%, conversion of DMA = 0%).

a

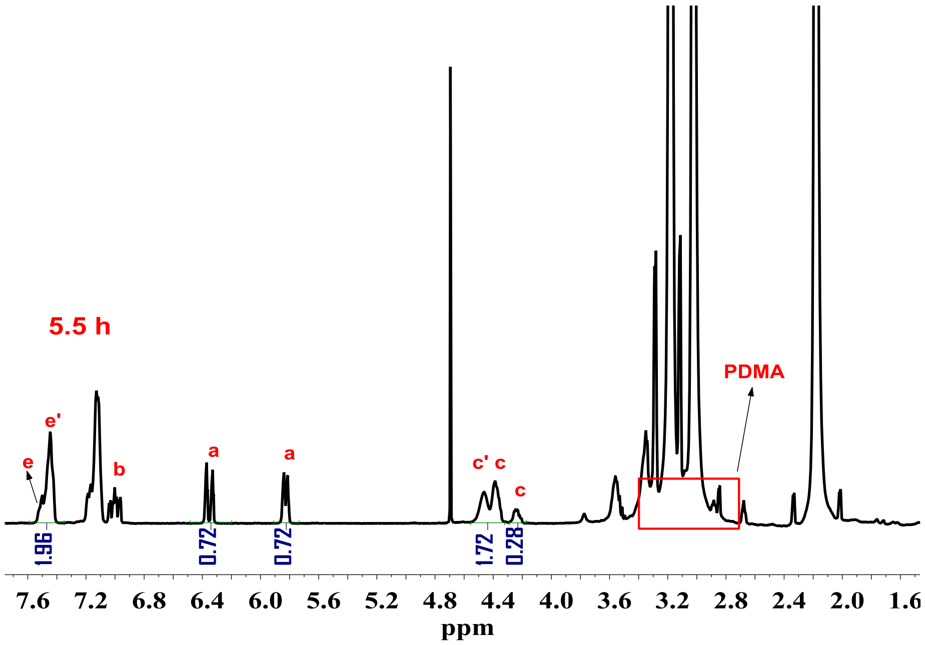

b

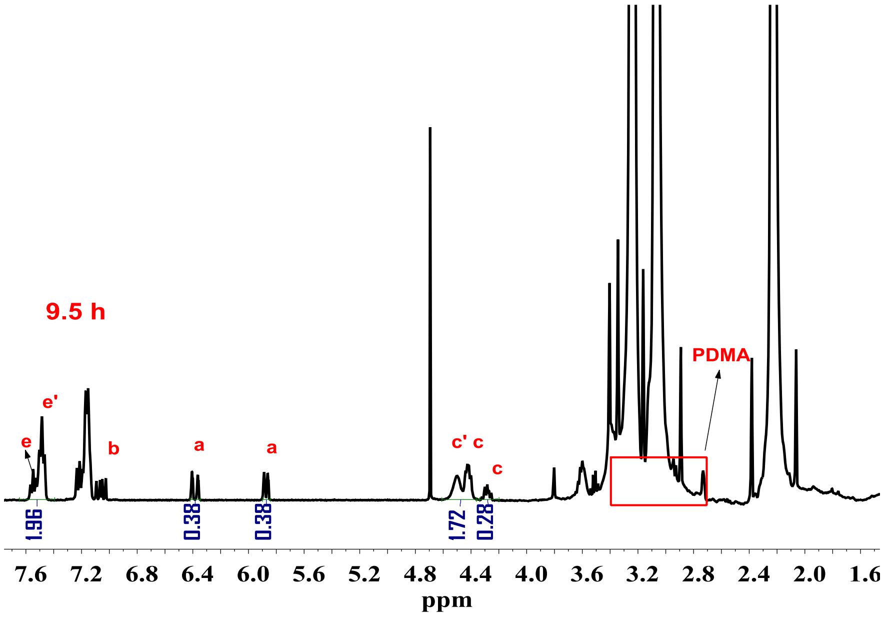

Supplementary Figure 5 ^{1}H–NMR trace showing the ON/OFF switch during the formation of ABA copolymer: a) 5.5 h after polymerization (4.5 h heating + 1 h irradiation; conversion of POMT = 72%, conversion of DMA = 28%). b) 9.5 h after polymerization (4.5 h heating + 5 h irradiation; conversion of POMT = 72%, conversion of DMA = 62%).

The absorption of trithiocarbonate is at 453 nm

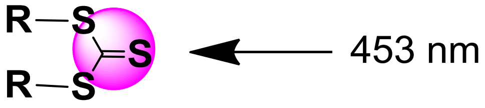

The absorption of trithiocarbonate neighbored to POMT units is at 438 nm.

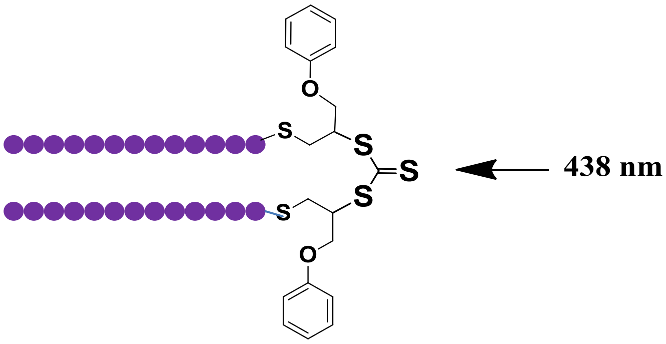

The absorption of trithiocarbonate neighbored to DMA units is at 433 nm.

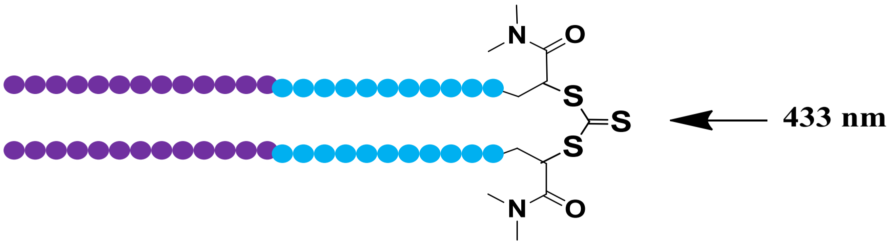

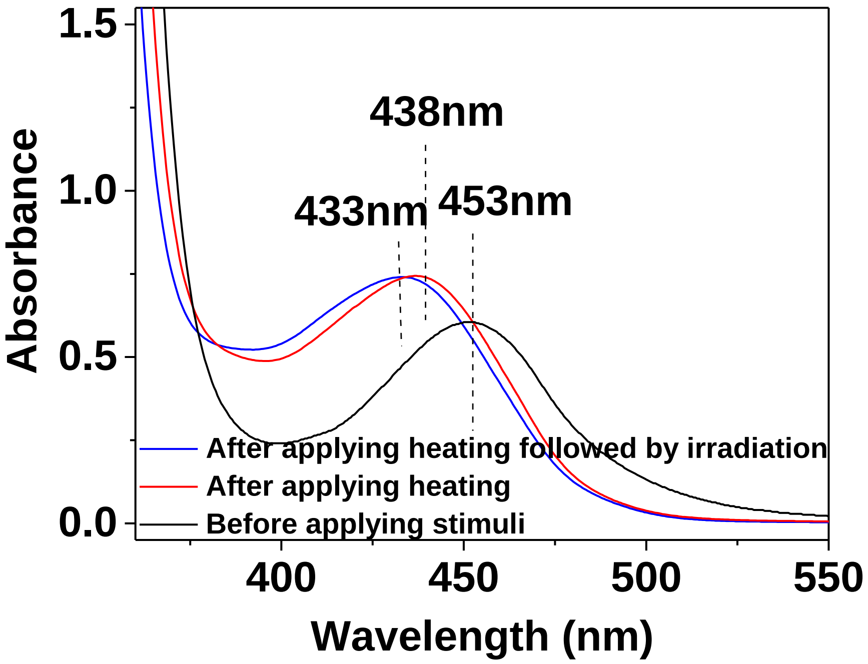

Supplementary Figure 6 UV−vis characteristic absorption curves of trithiocarbonate containing molecules or macromolecules via heating followed by irradiation.

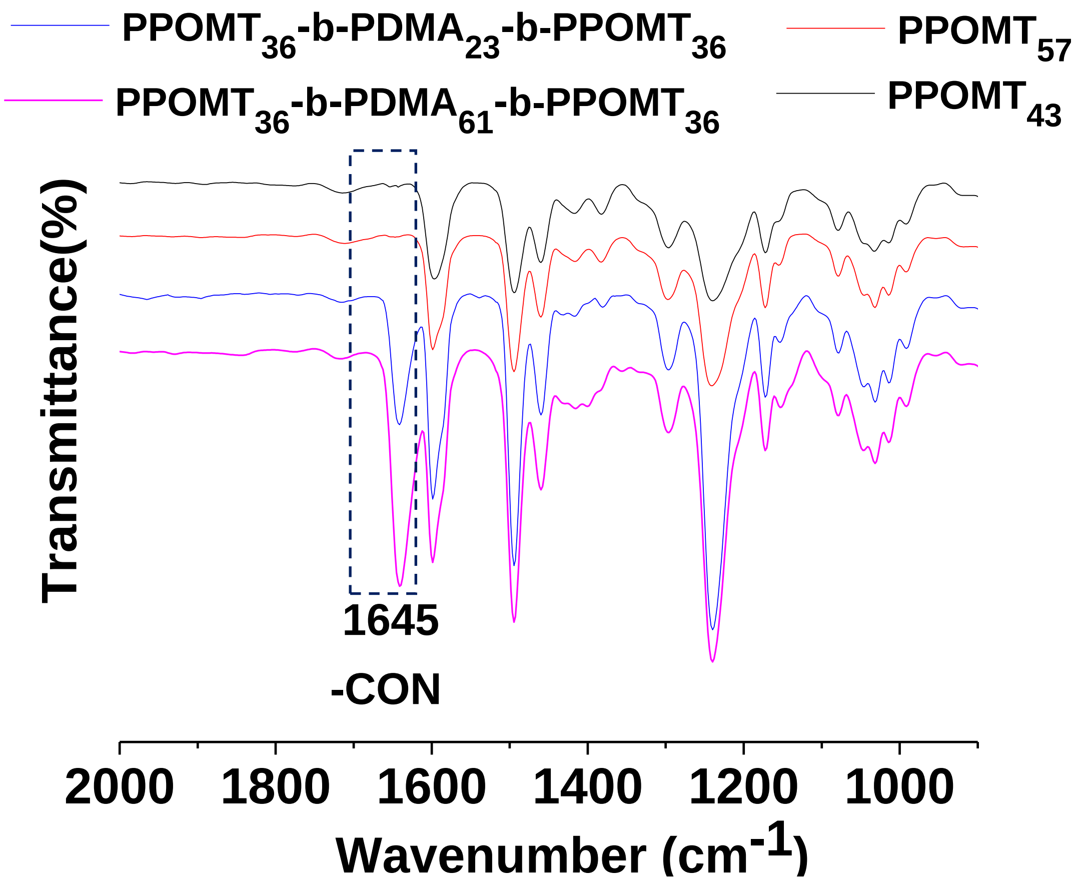

Supplementary Figure 7 FI–IR spectra of homopolymers or copolymer at each conversion during the copolymerization process via heating followed by irradiation (forming ABA-type triblock copolymer).

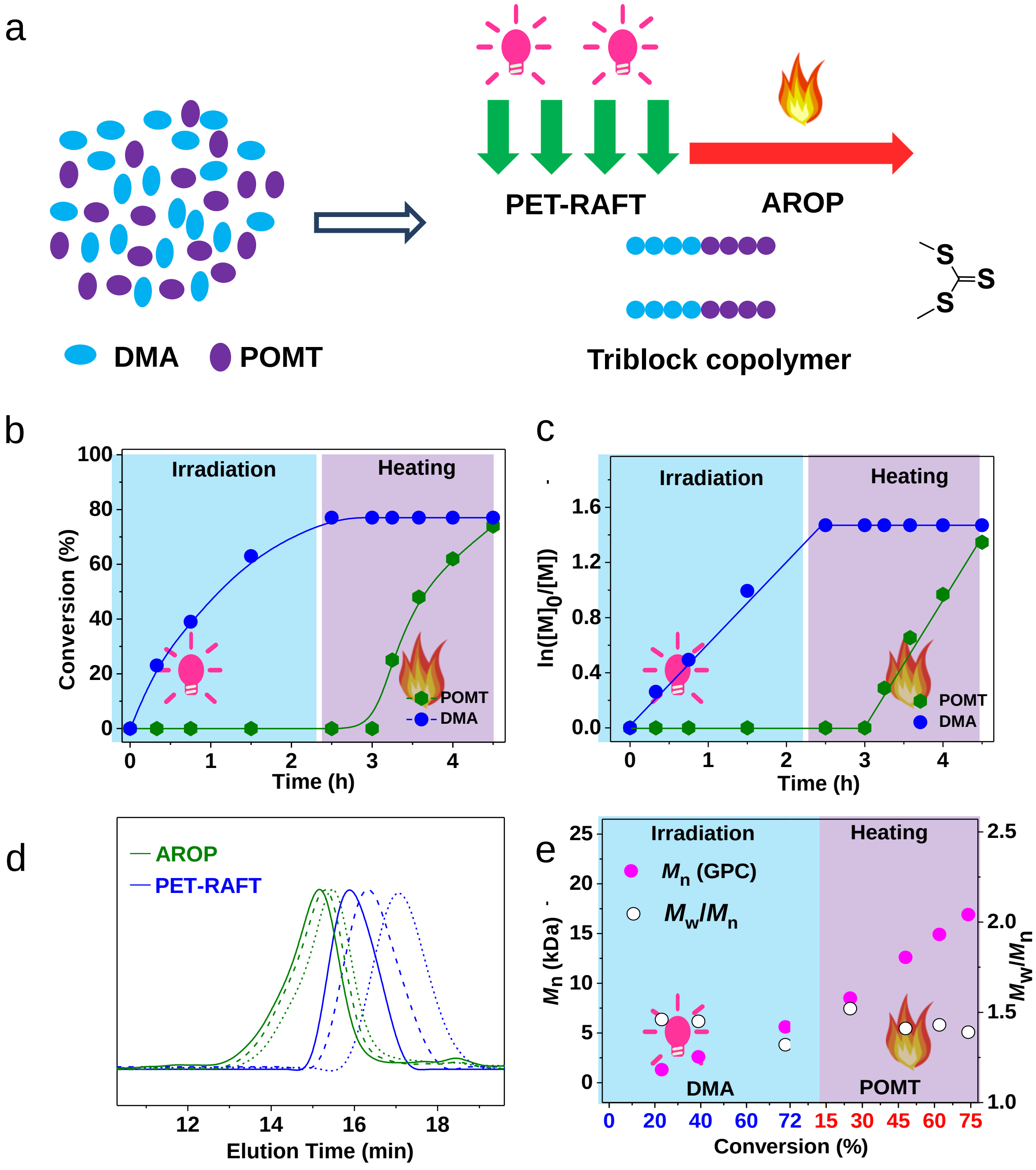

Supplementary Figure 8 Synthesis of BAB triblock copolymer by irradiation followed by heating: a) Schematic diagram of the polymerization process. b) Monomer conversion versus time. c) $\ln([M]_0/[M])$ versus reaction time. d) SEC curves for resulting polymers at different polymerization times under irradiation followed by heating. e) Molecular weight and polydispersity versus monomer conversion under irradiation followed by heating.

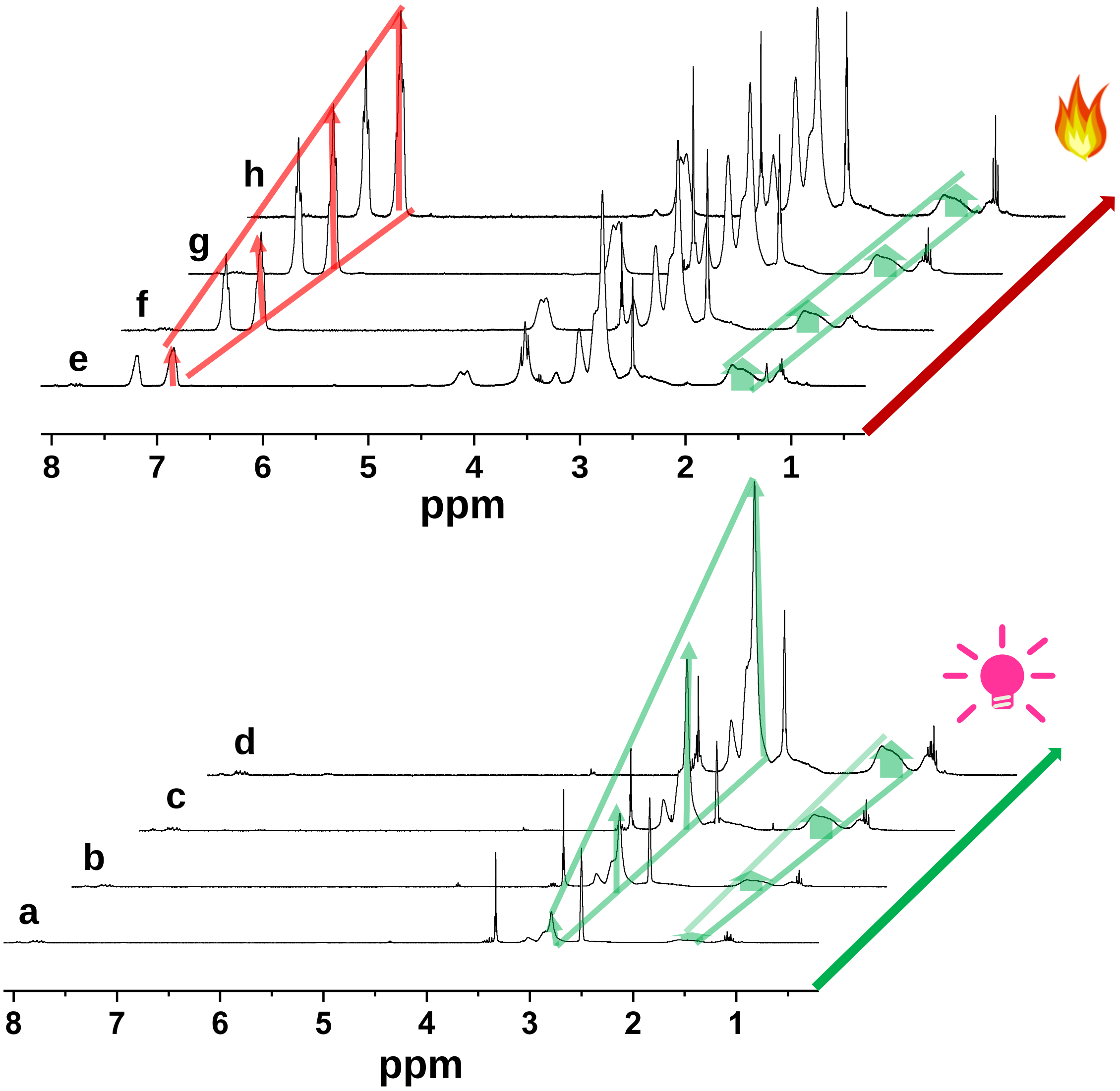

Supplementary Figure 9 ^{1}H–NMR spectra of the polymers produced at different conversion under irradiation followed by heating: a) $PDMA_{23}$. b) $PDMA_{39}$. c) $PDMA_{63}$. d) $PDMA_{77}$. e) $PDMA_{38}$-$PPOMT_{25}$-$PDMA_{39}$. f) $PDMA_{38}$-$PPOMT_{48}$-$PDMA_{39}$. g) $PDMA_{38}$-$PPOMT_{62}$-$PDMA_{39}$. h) $PDMA_{38}$-$PPOMT_{74}$-$PDMA_{39}$.

a

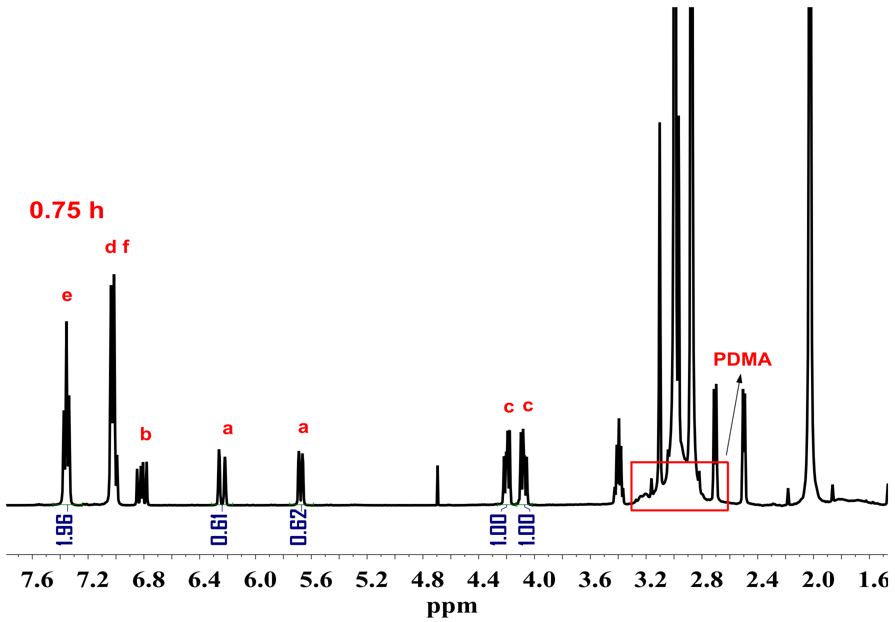

b

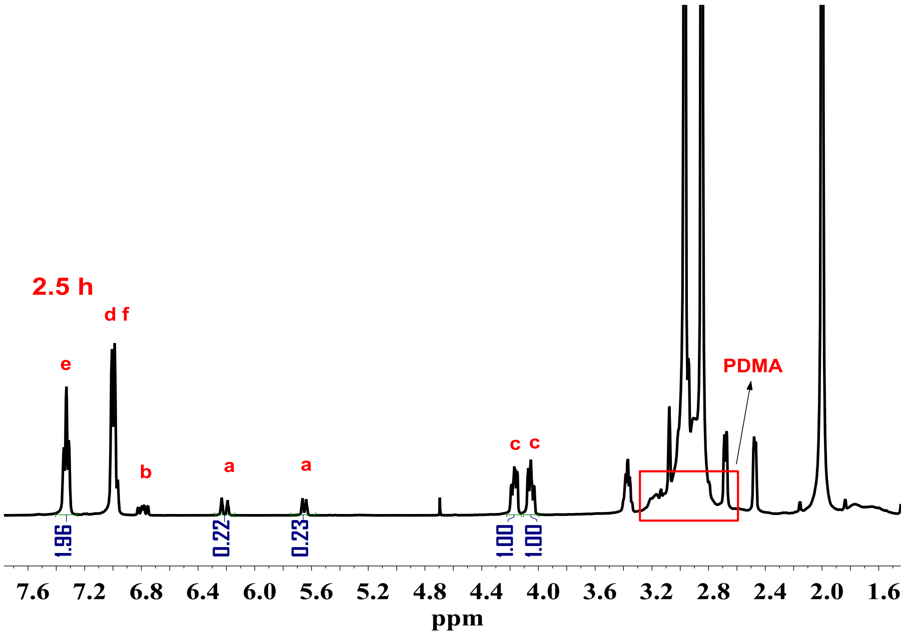

Supplementary Figure 10 ^{1}H–NMR trace showing the ON/OFF switch during the formation of BAB copolymer: a) 0.75 h after polymerization (0.75 h irradiation + 0 h heating; conversion of POMT = 0%, conversion of DMA = 38%). b) 2.5 h after polymerization (2.5 h irradiation + 0 h heating; conversion of POMT = 0%, conversion of DMA = 77%).

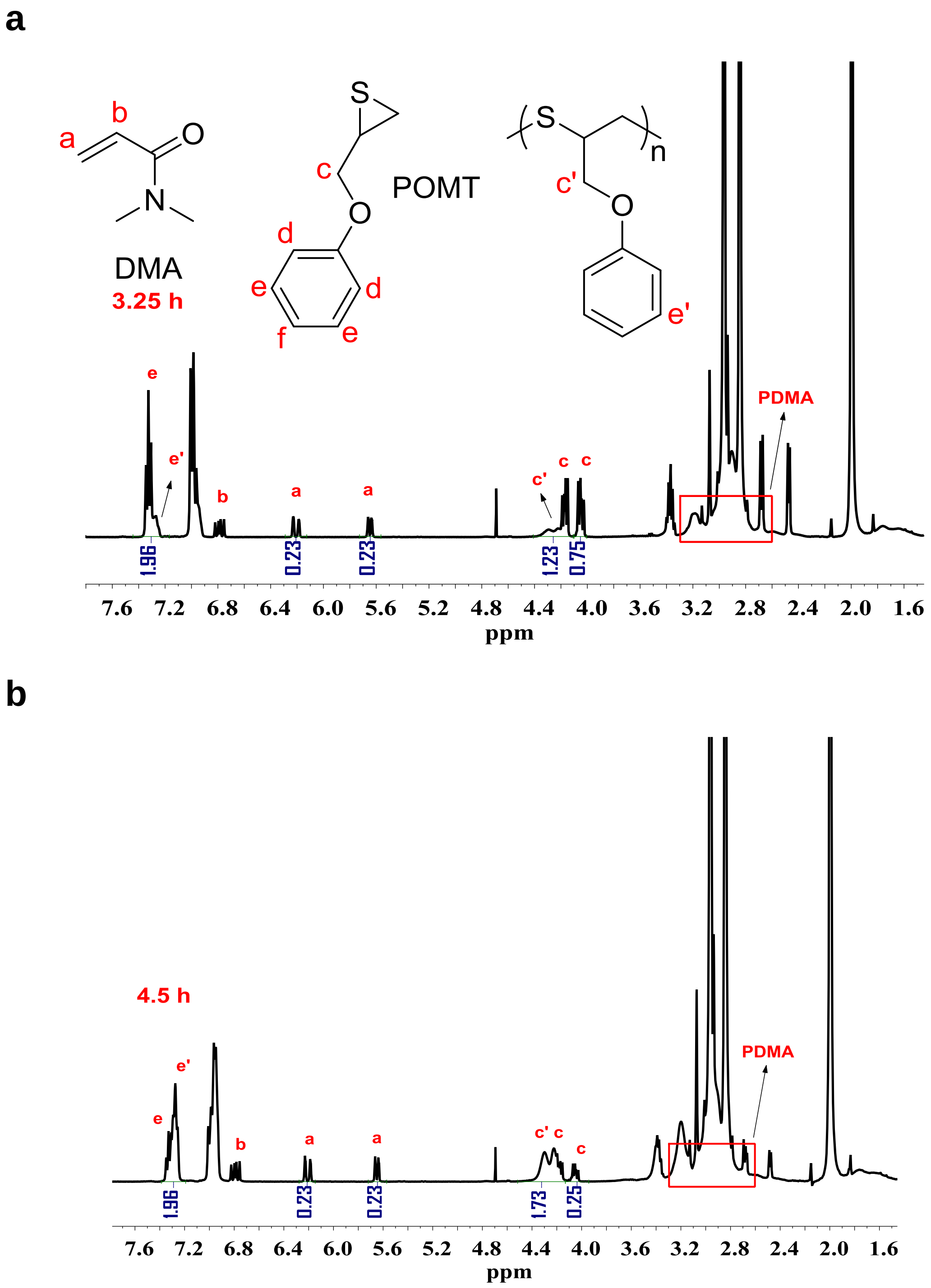

Supplementary Figure 11 ^{1}H–NMR trace showing the ON/OFF switch during the formation of BAB copolymer: a) 3.25 h after polymerization (2.5 h irradiation + 1.25 h heating; conversion of POMT = 25%, conversion of DMA = 77%). b) 4.5 h after polymerization (2.5 h irradiation + 2 h heating; conversion of POMT = 75%, conversion of DMA = 77%).

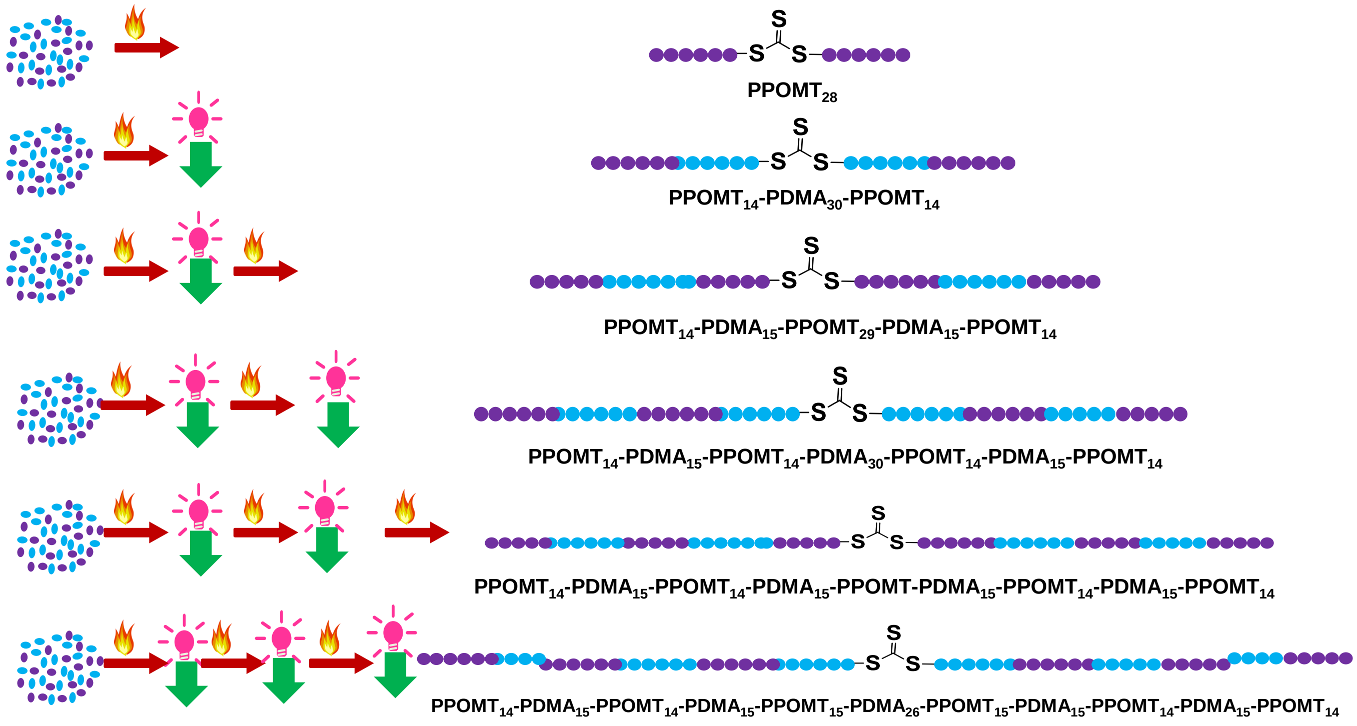

Supplementary Figure 12 The structures of multiblock copolymers after applying each heating and irradiation.

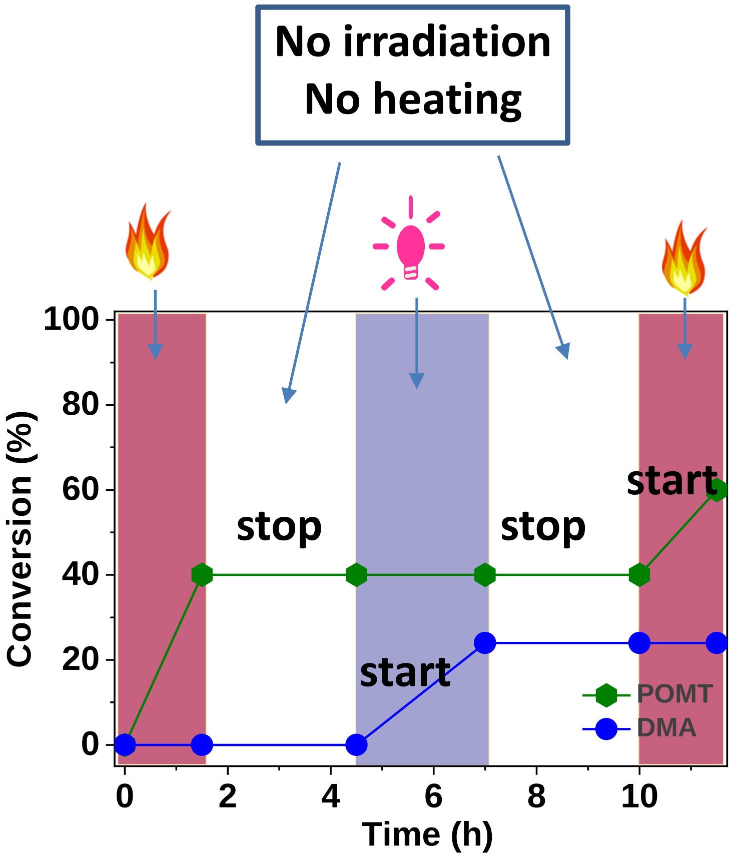

Supplementary Figure 13 The experiment on the polymerization completely stopping and restarting.

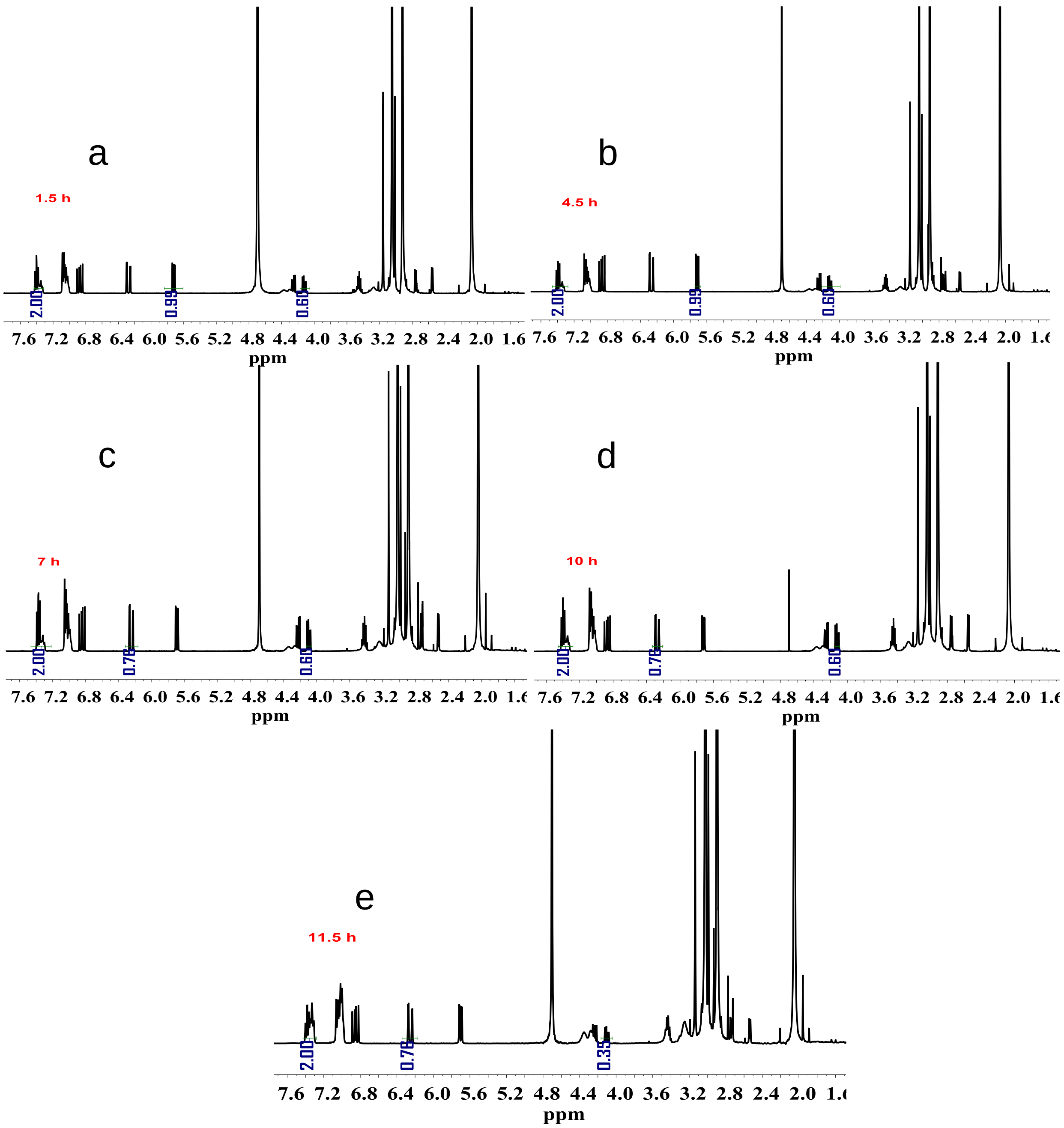

Supplementary Figure 14 ^{1}H–NMR trace of the polymerization process in the presence of light and heat and in absence of both light and heat: a) 1.5 h (1.5 h heating + 0 h irradiation; conversion of POMT=40%, conversion of DMA=0%). b) 4.5 h (1.5 h heating + 0 h irradiation + 3 h absence of light and heat; conversion of POMT=40%, conversion of DMA=0%). c) 7 h (1.5 h heating + 2.5 h irradiation + 3 h absence of light and heat; conversion of POMT=40%, conversion of DMA=24%). d) 10 h (1.5 h heating + 2.5 h irradiation + 6 h absence of light and heat; conversion of POMT=40%, conversion of DMA=24%). e) 11.5 h (3 h heating + 2.5 h irradiation + 6 h absence of light and heat; conversion of POMT=65%, conversion of DMA=24%).

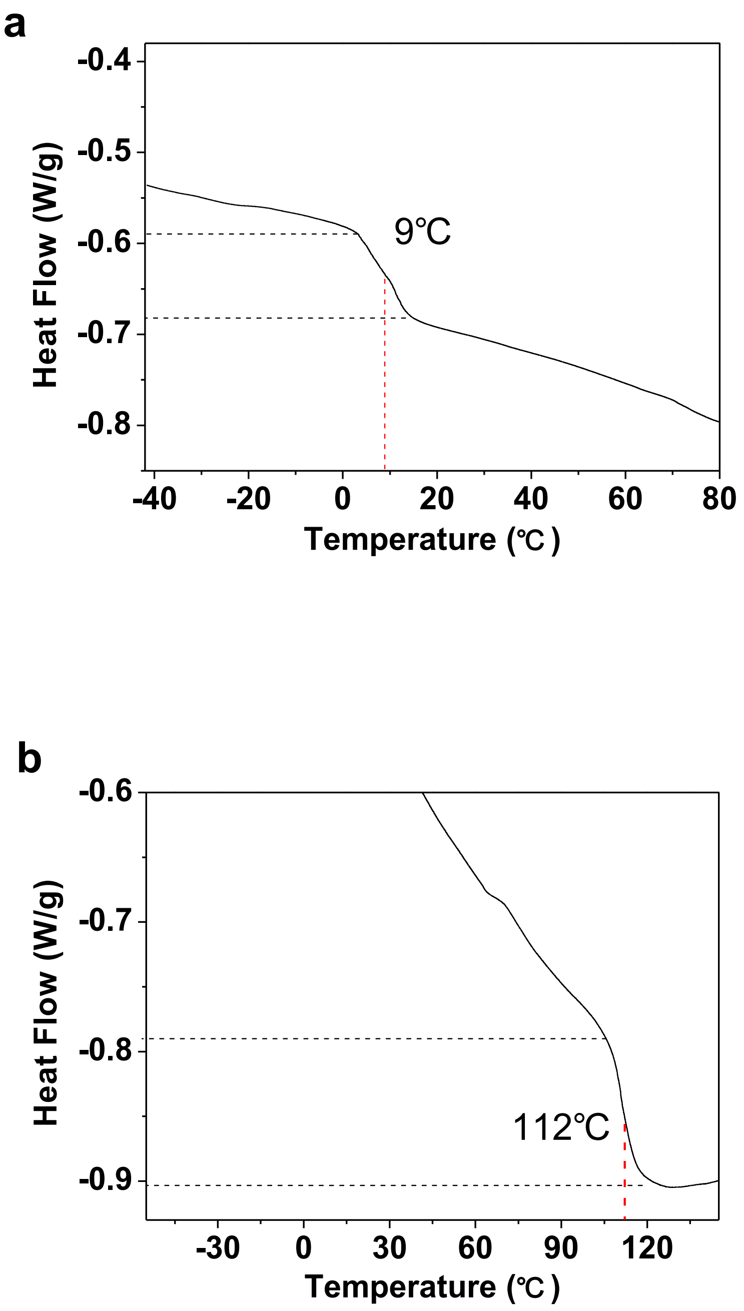

Supplementary Figure 15 Glass transition temperatures measured by DSC: a) Homopolymer PPOMT. b) Homopolymer PDMA.

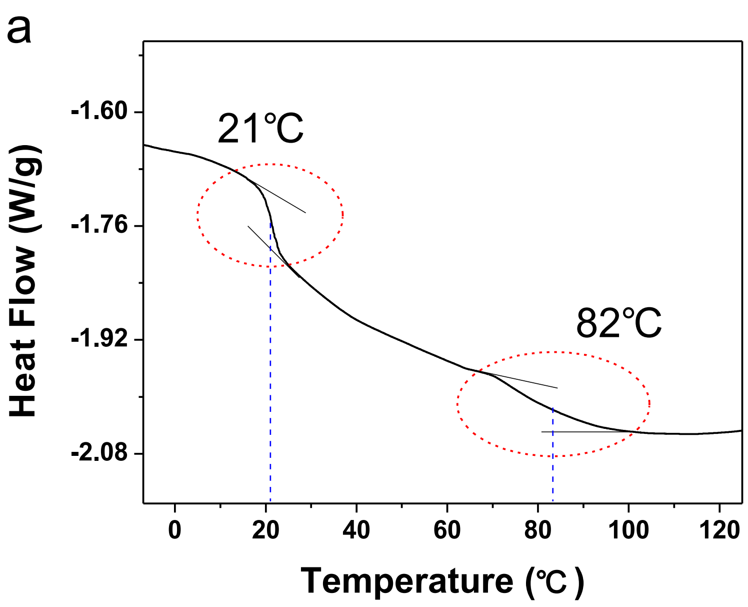

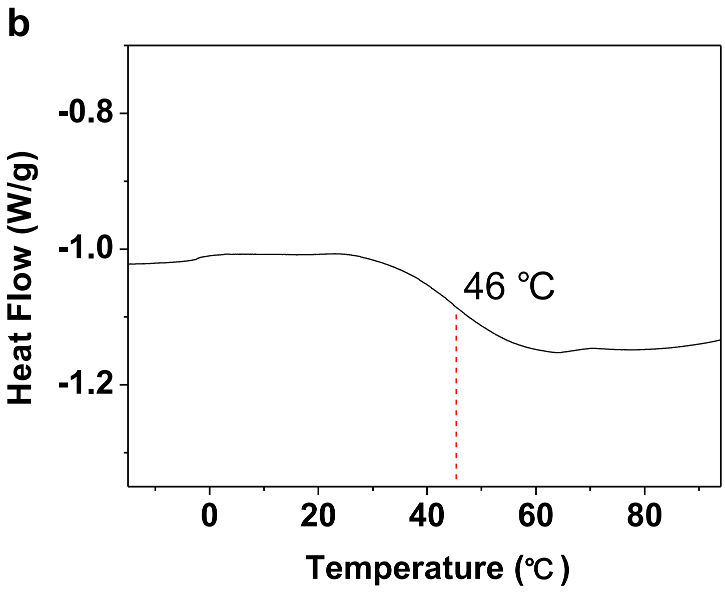

Supplementary Figure 16 Glass transition temperatures measured by DSC: a) The ABA triblock copolymer. b) The undecablock copolymer.

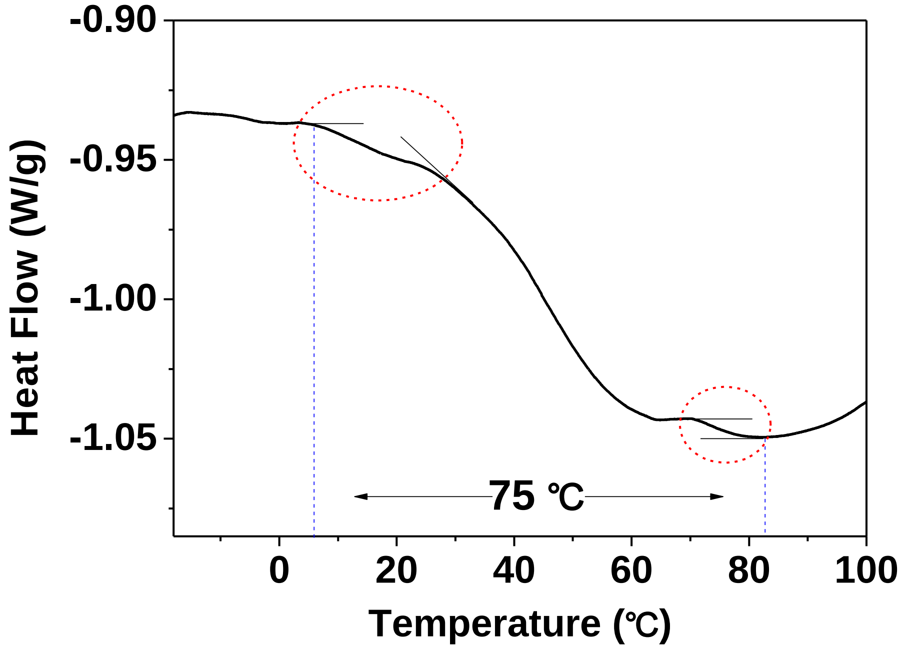

Supplementary Figure 17 DSC curve of obtained symmetrically gradient undecablock copolymer.

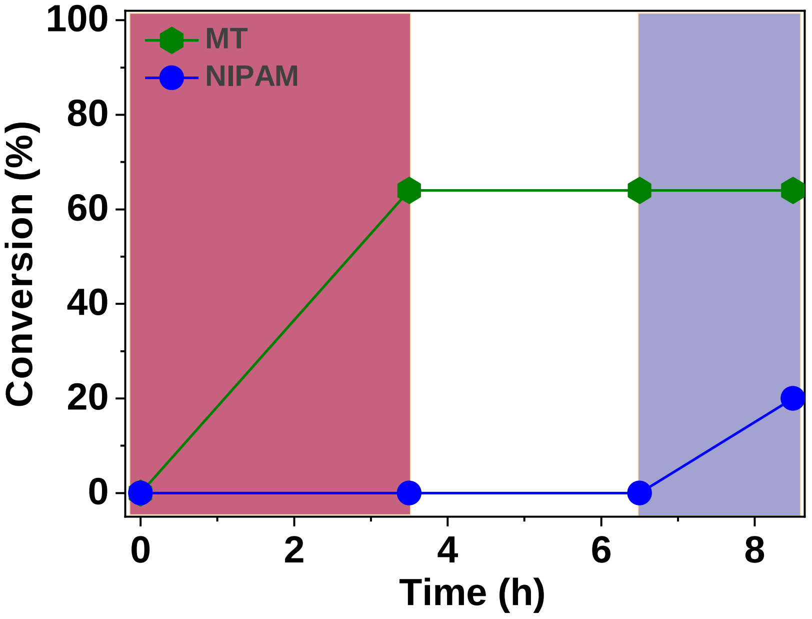

Supplementary Figure 18 The orthogonality of using 2-methylthiirane (MT) and *N*-isopropylacrylamide (NIPAM) as comonomers.

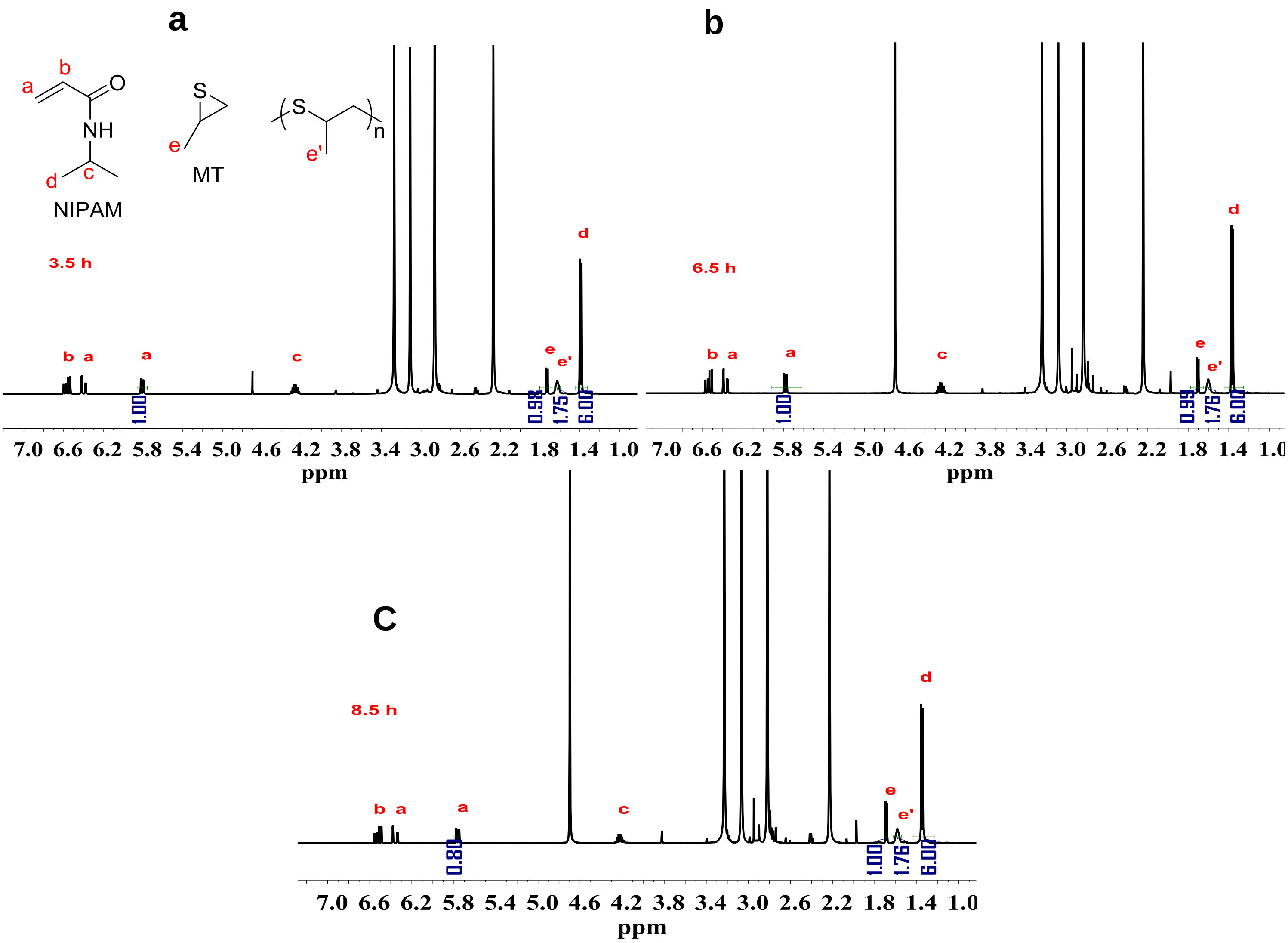

Supplementary Figure 19 ^{1}H–NMR trace of the orthogonal polymerization process using MT and NIPAM as model monomers: a) 3.5 h after polymerization (3.5 h heating + 0 h irradiation; conversion of MT = 64%, conversion of NIPAM = 0%). b) 6.5 h after polymerization (3.5 h heating + 3.0 h No irradiation and heating; conversion of MT = 64%, conversion of NIPAM = 0%). c) 8.5 h after polymerization (3.5 h heating + 3.0 h No irradiation and heating + 2.0 h irradiation; conversion of MT = 64%, conversion of NIPAM = 20%). Conversion (MT) = $(I_2)/(I_1+I_2) \times 100\%$, Conversion (NIPAM) = $(1-I_3) \times 100\%$. I_1 denotes the protons integral values of methyl at unreacted MT (δ = 1.74 ppm). I_2 denotes the protons integral values of methyl at PMT (δ = 1.63 ppm), I_3 denotes the protons integral values of methylene at unreacted NIPAM (δ = 5.82 ppm).

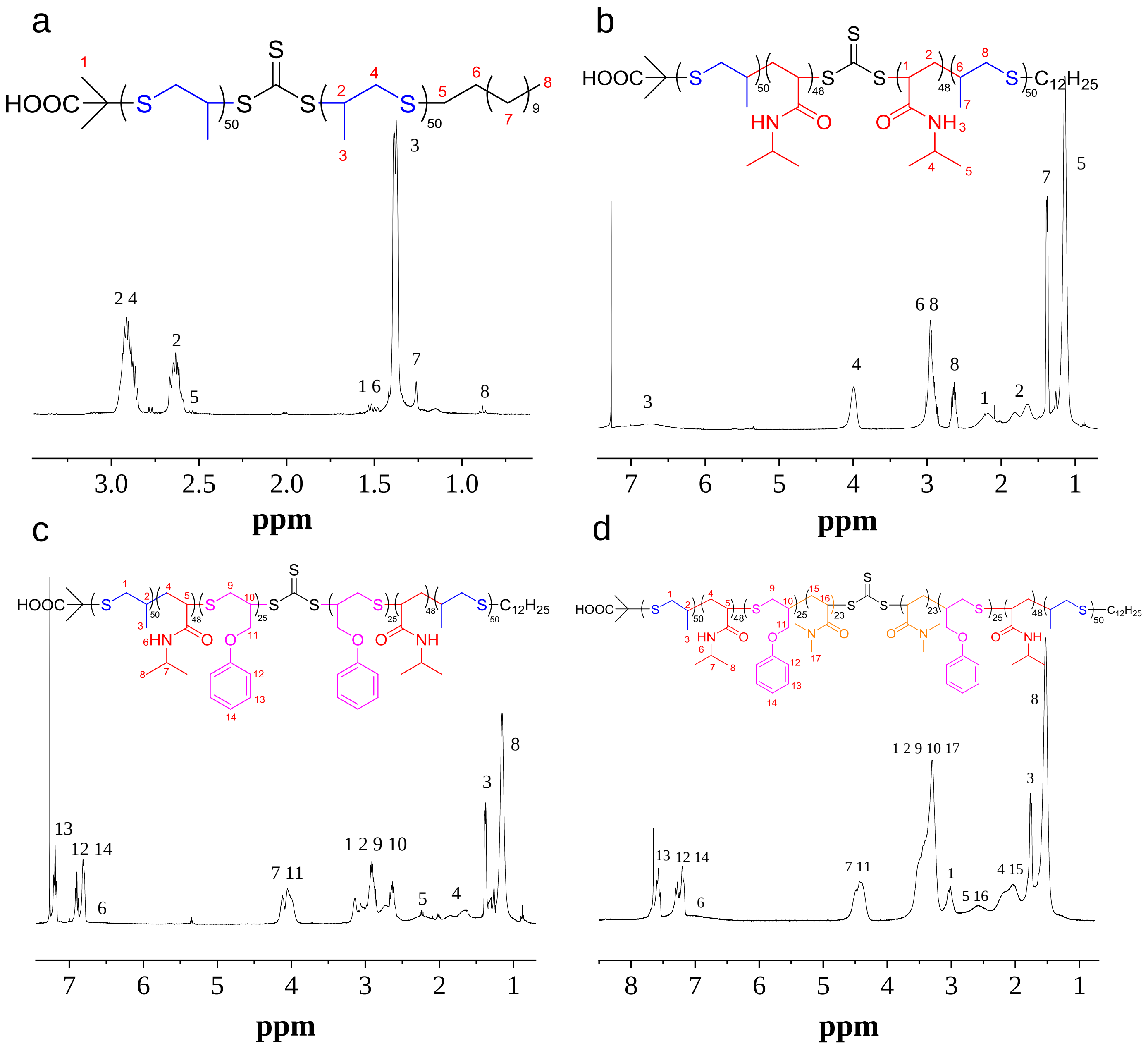

Supplementary Figure 20 ^{1}H–NMR spectrum of obtained PMT-*b*-PNIPAM-*b*-PPOMT-*b*-PDMA-*b*-PPOMT-*b*-PNIPAM-*b*-PMT heptablock quadricopolymer: a) PMT homopolymer in $CDCl_3$. b) PMT-b-PNIPAM-b-PMT triblock copolymer in $CDCl_3$. c) PMT-*b*-PNIPAM-*b*-PPOMT-*b*-PNIPAM-*b*-PMT pentablock copolymer in $CDCl_3$. d) ^{1}H NMR spectrum of obtained PMT-b-PNIPAM-b-PPOMT-b-PDMA-b-PPOMT-b-PNIPAM-b-PMT heptablock copolymer in $CDCl_3$.

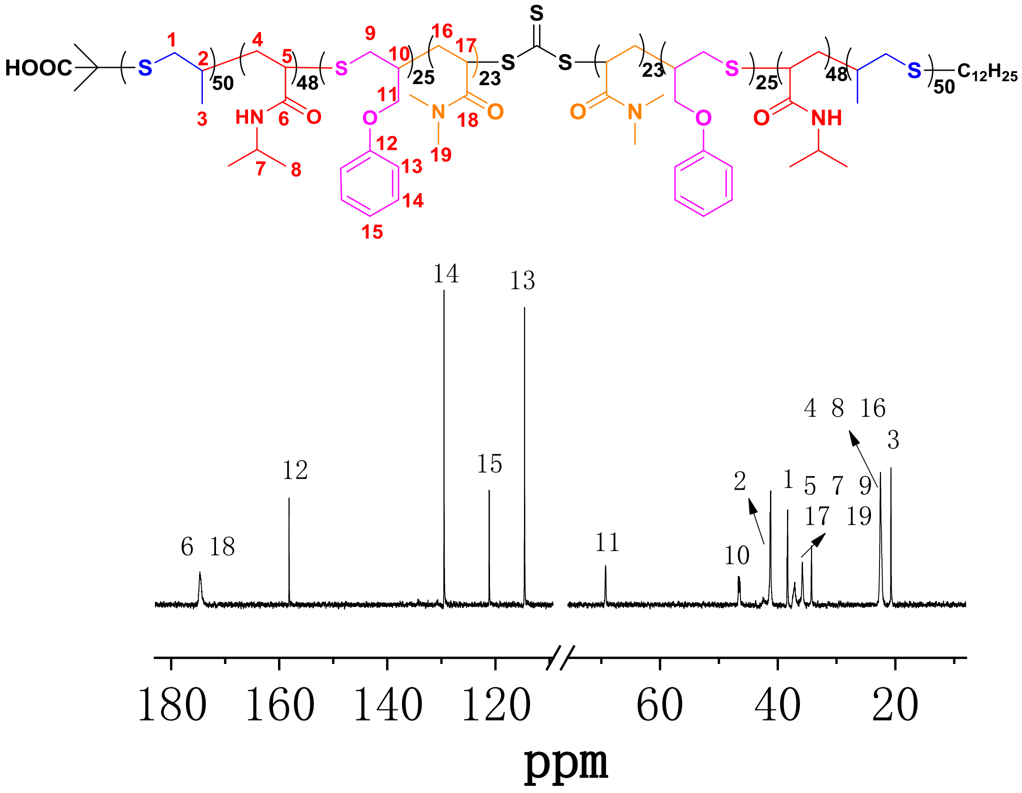

Supplementary Figure 21 ^{13}C–NMR spectrum of obtained PMT-*b*-PNIPAM-*b*-PPOMT-*b*-PDMA-*b*-PPOMT-*b*-PNIPAM-*b*-PMT heptablock quadricopolymer.

.

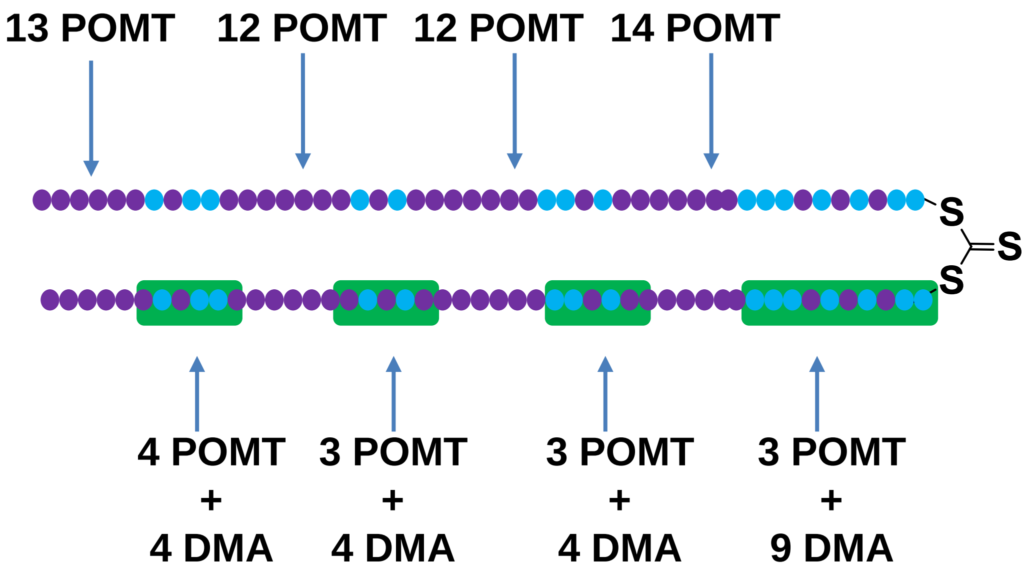

Supplementary Figure 22 Probable microstructure obtained via continuous heating combined with intermittent irradiation during copolymerization.

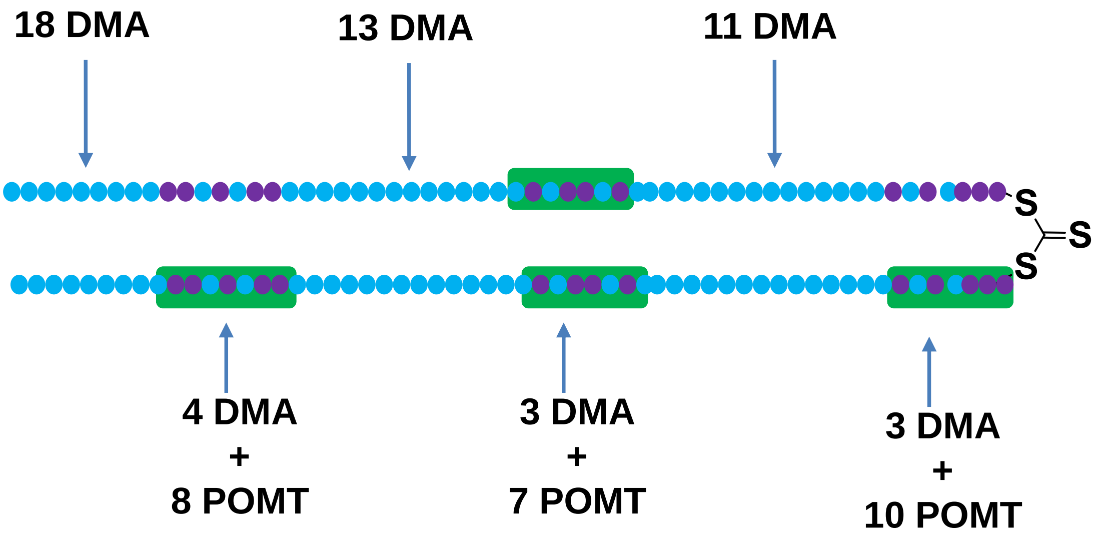

Supplementary Figure 23 Probable microstructure obtained via continuous irradiation combined with intermittent heating during copolymerization.

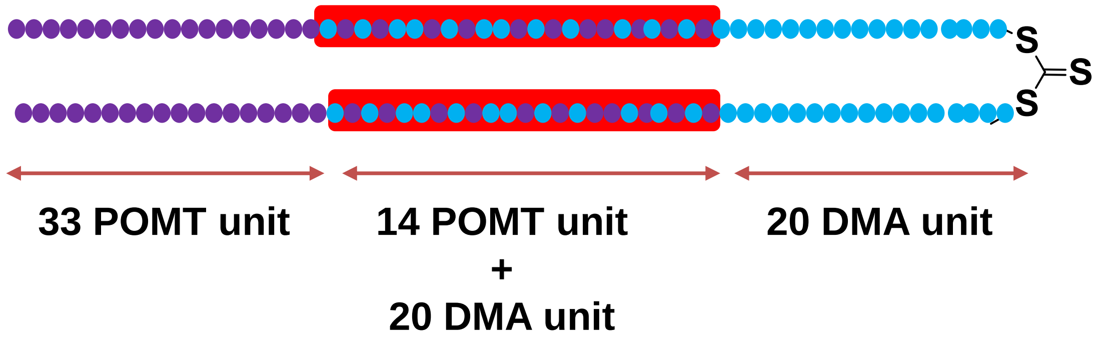

Supplementary Figure 24 Probable microstructure obtained via sequential heating, heating integrated with irradiation, and irradiation.

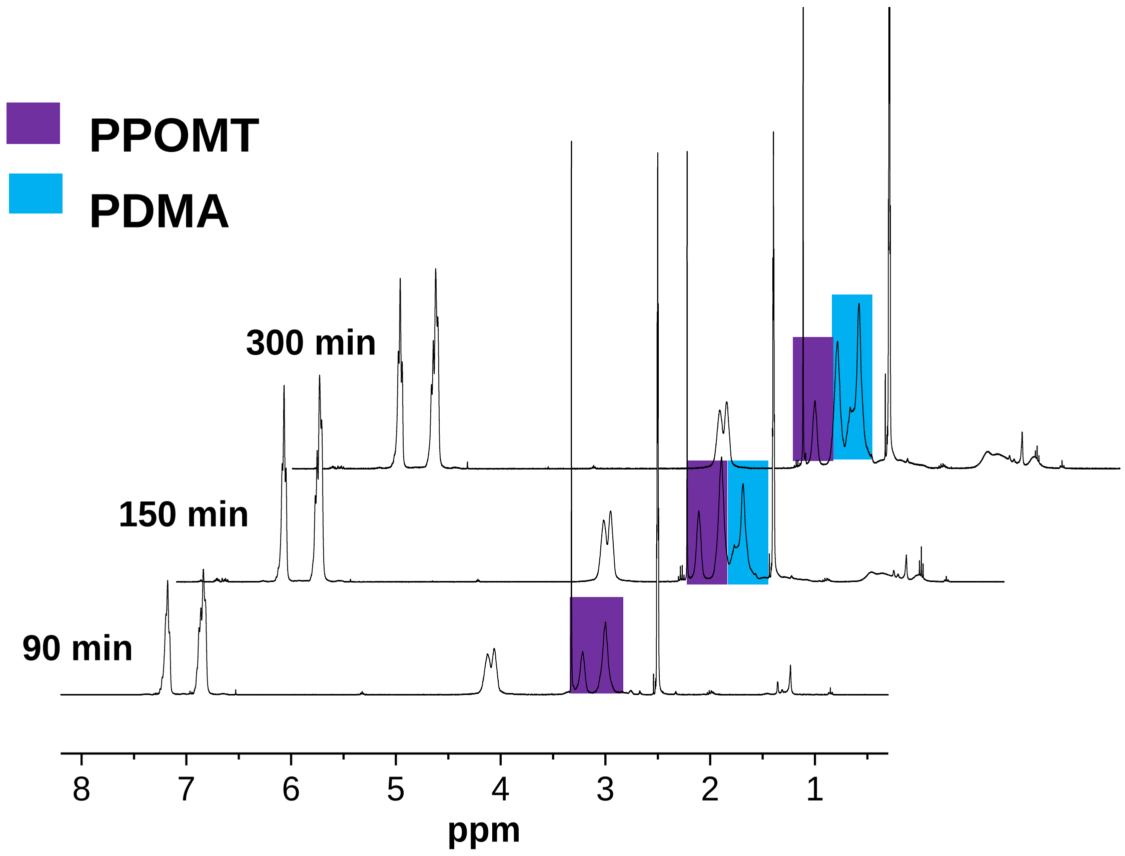

Supplementary Figure 25 ^{1}H NMR spectra of copolymers formed at different polymerization time via sequential heating, heating integrated with irradiation, and irradiation.

Supplementary Methods

Chemicals

Dimethylacetamide (DMAc, 99.8%, extra dry, with molecular sieves, water < 50 ppm) was purchased from Aladdin. 2-(Phenoxymethyl) oxirane (98%), tetraphenylphosphonium chloride (97%), tricaprylylmethylammonium chloride (97%) and tetrabutylammonium hydrogen sulfate (99%) were purchased from Energy Chemical. Chloroform-d (99.8 atom % D), deuterium oxide (99.9 atom % D), DMSO-d_6 (99.8 atom % D) and tris[2-phenylpyridinato-C2,*N*]iridium(III) (99%) were purchased from Sigma-Aldrich. Carbon disulfide (99%) was purchased from Adamas-Beta. 2-Methyloxirane (98%), 1-dodecanethiol (98%), sodium sulfate anhydrous (99%), potassium carbonate anhydrous (99.5%), potassium thiocyanate (99%), n-hexane (97%), ethyl acetate (99.5%), methanol (99.7%), chloroform (99%), acetone (99.5%) and diethyl ether (99.7%) were purchased from Sinopharm Chemical Reagent Co. Ltd. *N*,*N*-Dimethylacrylamide (DMA, Sigma-Aldrich, 99%) was purified by small aluminum oxide (basic) chromatography to remove inhibitor. *N*-Isopropylacrylamide (NIPAM, TCI Chemical, 99%) was purified by recrystallization. Photocatalyst tris[2-phenylpyridinato-C2,*N*]iridium(III) was dissolved in DMAc to obtain 50 mM stock solution before use. All reagents were used as received unless otherwise stated.

Characterizations

All NMR spectra were recorded on a Bruker NMR spectrometer (resonance frequency of 400 MHz for ^{1}H NMR and 100 MHz for ^{13}C NMR) operated in the Fourier transform mode. The samples were dissolved in deuterium oxide, chloroform-d or DMSO-d6 with tetramethylsilane (TMS) as an internal reference. Molecular weights and molecular weight distributions were measured by using a Waters 150C gel permeation chromatograph (GPC) equipped with microstyragel columns and an RI

2414 detector at 30 ℃. LiBr/DMF (0.1%, w/w) solution with a flow rate of 1.0 mL/min was used as eluent. The molecular weights were calibrated against monodispersed polystyrene standards. Differential scanning calorimetry (DSC) thermograms were measured on a TA Q2000 differential scanning calorimeter instrument in aluminum pans with a heating or cooling rate of 10 ℃/min under a flowing nitrogen atmosphere from -50 ℃ to 180 ℃. All T_g values were obtained from the second scan after removing the thermal history [1].

Synthesis of monomers and trithiocarbonate

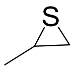

MT

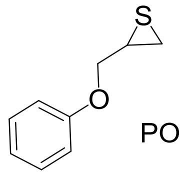

POMT

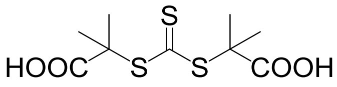

BDMAT

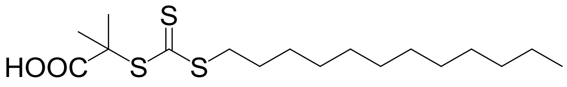

DDMAT

Synthesis of 2-methylthiirane (MT). To a solution of 2-methyloxirane (29 g, 500 mmol) in distilled water (150 mL), was added potassium thiocyanate (194 g, 2 mol). After the mixture was stirred at room temperature for 24 h, the organic phase was separated and dried over anhydrous sodium sulfate. The product 2-methylthiirane as colorless liquid was obtained by distillation. Yield was 83%. ^{1}H NMR spectrum (400 MHz, $CDCl_3$): δ 1.531 (d, 3H), δ 2.139 (d, 1H), δ 2.526 (d, 1H), δ 2.919 (m, 1H).

Synthesis of 2-(phenoxymethyl) thiirane (POMT). To a suspension of 2-(phenoxymethyl)oxirane (9.0 g, 60 mmol) in distilled water (30 mL), was added potassium thiocyanate (23.2 g, 240 mmol). After the mixture was stirred at 40 ℃ for 24 h, the organic phase was separated and the aqueous phase was extracted with diethyl ether (2 × 30 mL). The combined organic phases were dried over anhydrous

sodium sulfate. Further purification was carried out by silica gel column chromatography using hexane/ethyl acetate (v/v, 9:1). The product was obtained as colorless viscous oil. Yield was 75%. ^{1}H NMR spectrum (400 MHz, $CDCl_3$): δ 2.349 (d, 1H), δ 2.632 (d, 1H), δ 3.307 (m, 1H), δ 3.961 (m, 1H), δ 4.224 (m, 1H), δ 6.959 (m, 3H), δ 7.325 (m, 2H).

Synthesis of S,S'-bis(α,α'-dimethyl-α"-acetic acid) trithiocarbonate (BDMAT). Carbon disulfide (13.8 g, 0.18 mol), chloroform (58.8 g, 0.45 mol), acetone (26.3 g, 0.45 mol), tetrabutylammonium hydrogen sulfate (1.2 g, 3.6 mmol) and *n*-hexane (60 mL) were mixed in a 250 mL bottom flask. The mixture was cooled in an ice bath. Sodium hydroxide (50 %, 101 g) was added dropwise in 60 min. Then the reaction was left to stir overnight. Water (500 mL) was added to the reaction mixture, followed by slow addition of concentrated HCl (60 mL) while stirring to acidify the aqueous layer under an argon atmosphere. The precipitate was filtered, rinsed thoroughly with water, and dried under vacuum to yield brown-colored solid. The solid was purified by recrystallization in acetone/hexane (4/1, v/v) thrice to obtain yellow crystal. Yield was 37%. ^{1}H NMR spectrum (400 MHz, $CDCl_3$): δ 1.69 (s, 12H).

Synthesis of S-1-dodecyl S'-(α,α-dimethylacetic acid) trithiocarbonate (DDMAT). 1-Dodecanethiol (20.2 g, 0.1 mol), acetone (58 g, 1 mol) and tricaprylylmethylammonium chloride (1.63 g, 0.004 mol) were mixed in a 500 mL bottom flask. The mixture was cooled in an ice bath. Sodium hydroxide (50 %, 8.4 g) was added dropwise and stirred for an additional 15 minutes. The reaction mixture turned very viscous as a white precipitate formed. Carbon disulfide (7.6 g, 0.1 mol) in acetone (10.4 g, 0.18 mol) was added dropwise to the reaction vessel. The reaction mixture turned yellow. The mixture was subsequently stirred for an additional 10 minutes after the addition completed. Chloroform (18 g, 0.15 mol) was then added in one portion, followed by dropwise addition of sodium hydroxide (50 %, 40 g) in 30 min. The reaction was left to stir overnight and was observed to

turn red. Water (150 mL) was added to the reaction vessel, followed by slow addition of concentrated HCl (25 mL) while stirring. The solid formed was collected via filtration and stirred in isopropanol (250 mL). The remaining precipitate was filtered off and the filtrate was collected. The solvent was evaporated off. The product was purified by recrystallization in *n*-hexane thrice to yield a yellow crystal. Yield was 45%. ^{1}H NMR spectrum (400 MHz, $CDCl_3$): δ 0.88 (t, 3H), δ 1.25 - 1.72 (m, 26H), δ 3.28 (t, 2H), δ 10.95 (s, 1H).

Polymerization via programed process

AROP of thiirane under varying temperatures.

BDMAT (12.8 mg, 0.045 mmol), POMT (747 mg, 4.5 mmol) and tetraphenylphosphonium chloride (5.6 mg, 0.015 mmol) were dissolved in 3 mL DMAc, and then transferred into a transparent glass tube with rubber plug. After three freeze-pump-thaw cycles, the tube was sealed and immersed in an oil bath at 40, 50, 60, 70 ℃ or placed at room temperature (20 ℃). Conversions were recorded by ^{1}H NMR spectra after suitable time interval with NMR tubes adapted with coaxial inserts after appropriate time intervals. D_2O was in the inner of the concentric capillary tube, while the mixed solution in the outer capillary tube.

Switch ON/OFF experiment of BDMAT mediated AROP of POMT.

BDMAT (12.8 mg, 0.045 mmol), POMT (747 mg, 4.5 mmol) and tetraphenylphosphonium chloride (5.6 mg, 0.015 mmol) were dissolved in 3 mL DMAc and transferred into a transparent glass tube with rubber plug. After three freeze-pump-thaw cycles, the tube was sealed and immersed in an oil bath at 50 ℃. After 1h's heating, the tube was quenched into ice water for 2 min and placed at room temperature for 1 h. Then the mixture was repeatedly heated and cooled. Conversions were recorded by ^{1}H NMR spectra after suitable time interval.

Switch ON/OFF experiment of BDMAT mediated PET-RAFT polymerization.

DMA (594 mg, 6 mmol), BDMAT (16.8 mg, 0.06 mmol) and photocatalyst ($Ir(ppy)_3$, 12 μL, 10 ppm) were dissolved in DMAc to obtain 4 mL solution and transferred into a glass tube with rubber plug. After three freeze-pump-thaw cycles, the tube was sealed and irradiated by 5W blue LED strip. After 45 min's irradiation, turn off the light and put the tube in the dark for 30 min. Then the LED strip was repeatedly turned on and turned off. Conversions were recorded by ^1H NMR spectra after suitable time interval.

Copolymerization via heating followed by irradiation (forming ABA triblock copolymer).

DMA (891 mg, 9 mmol), POMT (1494 mg, 9 mmol), BDMAT (25.5 mg, 0.09 mmol), phhotocatalyst (27 μL, 15 ppm) and tetraphenylphosphonium chloride (11 mg, 0.03 mmol) were dissolved in DMAc to obtain 6 mL solution, which was further transferred into a glass tube with rubber plug. After three freeze-pump-thaw cycles, the tube was sealed and immersed in an oil bath at 60 ℃. After suitable time intervals, moderate solutions were taken out by injector. Conversions of POMT were calculated by ^1H NMR spectra. After 4.5 h's heating (conversion of POMT reaching 72%), the tube was quickly cooled to room temperature and irradiated by 5W blue LED strip. Similarly, after suitable time intervals, moderate solutions were taken out by injector, and conversions of DMA were calculated by ^1H NMR spectra. All the reaction mixtures with different reaction times were precipitated into diethyl ether several times and the products as light yellow solids were obtained after dried in vacuum.

Copolymerization via irradiation followed by heating (forming BAB triblock copolymer).

DMA (891 mg, 9 mmol), POMT (1494 mg, 9 mmol), BDMAT (25.5 mg, 0.09 mmol), phhotocatalyst (27 μL, 15 ppm) and tetraphenylphosphonium chloride (11 mg, 0.03 mmol) were dissolved in DMAc to obtain 6 mL solution, which was further

transferred into a glass tube with rubber plug. After three freeze-pump-thaw cycles, the tube was sealed and irradiated by 5W blue LED strip. After suitable time intervals, moderate solutions were taken out by injector, and conversions of DMA were calculated by ^{1}H NMR spectra. After 2.5 h's irradiation (conversion of DMA reaching 77%), turn off the light source and the tube was immersed in an oil bath at 60 ℃. Similarly, after suitable time intervals, moderate solutions were taken out by injector, and conversions of POMT were calculated by ^{1}H NMR spectra. All the reaction mixtures with different reaction times were precipitated into diethyl ether several times and the products as light yellow solids were obtained after dried in vacuum.

The formation multiblock copolymers via multiple cycles of heating followed by irradiation.

DMA (891 mg, 9 mmol), POMT (1494 mg, 9 mmol), BDMAT (20.1 mg, 0.072 mmol), phhotocatalyst (45 μL, 25 ppm) and tetraphenylphosphonium chloride (11 mg, 0.03 mmol) were dissolved in DMAc to obtain 6 mL solution, which was further transferred into a glass tube with rubber plug. After three freeze-pump-thaw cycles, the tube was sealed and immersed in an oil bath at 60 ℃. After conversion of POMT reaching 23%, the tube was quickly cooled to room temperature and irradiated by 5W blue LED strip. Similarly, after conversion of DMA reaching 25%, the light was turned off and the tube was immersed into oil bath at 60 ℃ again. After conversion of POMT reaching 46%, the tube was cooled to room temperature and irradiated by blue LED strip again. Then after conversion of DMA reaching 49%, the light was turned off, and the tube was immersed into oil bath at 60 ℃ for the third time. After conversion of POMT reaching 70%, the tube was cooled to room temperature and irradiated by blue LED strip. All the reaction mixtures with different reaction times were precipitated into diethyl ether several times and the products as light yellow solids were obtained after dried in vacuum.

Preparing multiblock quadripolymer by the program of sequentially adding monomer and sequential heating and irradiation.

NIPAM (565 mg, 5 mmol), MT (370 mg, 5 mmol), DDMAT (18.2 mg, 0.05 mmol), phhotocatalyst (50 μL) and tetraphenylphosphonium chloride (18.7 mg, 0.05 mmol) were dissolved in DMAc to obtain 4 mL solution, which was further transferred into a glass tube with rubber plug. After three freeze-pump-thaw cycles, the tube was sealed and immersed in an oil bath at 60 °C. After 12h's heating, the tube was cooled to room temperature and irradiated by 5W blue LED strip for 24h. After each step, taken out 1.0 ml mixture by injector for further NMR analysis and precipitated into diethyl ether to get product. Then DMA (125 mg, 1.25 mmol) and POMT (207 mg, 1.25 mmol) were dissolved in 1 mL DMAc and added into above tube to obtain a homogeneous solution. The tube was sealed and immersed in oil bath at 60 ℃ again. After 24 h's heating, the tube was cooled to room temperature and irradiated by 5W blue LED strip for another 24 h. The mixture after each step was also taken out for further analysis.

Preparing copolymers with programmed sequence structure by using continuous heating with intermittent irradiation.

DMA (891 mg, 9 mmol), POMT (1494 mg, 9 mmol), BDMAT (16.8 mg, 0.06 mmol), photocatalyst (18 μL, 10 ppm) and tetraphenylphosphonium chloride (5.6 mg, 0.015 mmol) were dissolved in DMSO to obtain 6 mL solution, which was transferred into a glass tube with rubber plug. After three freeze-pump-thaw cycles, the tube was sealed and immersed in an oil bath at 45 ℃ for continuous heating. After appropriate time intervals, the tube was irradiated by 5W blue LED strip for 10 min each time combined with heating. All the reaction mixtures with different conversion were precipitated into diethyl ether several times and the products were obtained after dried in vacuum.

Preparing copolymers with programmed sequence structure by using continuous irradiation with intermittent heating.

DMA (891 mg, 9 mmol), POMT (996 mg, 6 mmol), BDMAT (16.8 mg, 0.06 mmol),

photocatalyst (9 μL, 5 ppm) and tetraphenylphosphonium chloride (5.6 mg, 0.015 mmol) were dissolved in DMSO to obtain 6 mL solution, which was further transferred into a glass tube with rubber plug. After three freeze-pump-thaw cycles, the tube was sealed and irradiated by 5W blue LED strip for 30 min. Then the tube was immersed in oil bath at 50 ℃ for 15 min while irradiation is still going on. The tube was quenched into ice water for 2 min and then irradiated by blue LED strip again for 45 min without heating. And later, the tube was immersed in oil bath at 50 ℃ for 20 min while irradiation going on. The tube was quenched into ice water for and irradiated by blue LED strip again for 60 min without heating. Lastly, the tube was immersed in oil bath at 50 ℃ for 20 min with blue LED irradiation. Reaction mixtures with different conversion were precipitated into diethyl ether several times and the products were obtained after dried in vacuum.

Copolymer with programmed sequence control via sequential heating, heating integrated with irradiation, and irradiation.

DMA (891 mg, 9 mmol), POMT (1494 mg, 9 mmol), BDMAT (16.8 mg, 0.06 mmol), photocatalyst (18 μL, 10 ppm) and tetraphenylphosphonium chloride (5.6 mg, 0.015 mmol) were dissolved in DMSO to obtain 6 mL solution, and transferred into a glass tube with rubber plug. After three freeze-pump-thaw cycles, the tube was sealed and immersed in an oil bath at 45 ℃ for 90 min. Then 5 W blue LED strip was turned on to irradiated the tube for 60 min with heating. Then the tube was quenched into ice water and irradiated by blue LED strip again for 150 min without heating. Reaction mixtures with different conversion were precipitated into diethyl ether several times and the products were obtained after dried in vacuum.

Copolymer with programmed sequence control via multiple cycles of sequential heating, heating integrated with irradiation, and irradiation.

DMA (891 mg, 9 mmol), POMT (1494 mg, 9 mmol), BDMAT (16.8 mg, 0.06 mmol), photocatalyst (27 μL, 15 ppm) and tetraphenylphosphonium chloride (5.6 mg, 0.015

mmol) were dissolved in DMSO to obtain 6 mL solution and transferred into a glass tube with rubber plug. After three freeze-pump-thaw cycles, the tube was sealed and immersed in an oil bath at 45 ℃ for 55 min. Then 10W blue LED strip was turned on to irradiated the tube for 15 min with heating. Then the tube was quenched into ice water and irradiated by blue LED strip again for 60 min without heating. This heating, combined heating and irradiation, irradiation cycles were repeated for other twice to get the conversions of POMT and DMA reach 76% and 75% respectively. Reaction mixtures with different conversion were precipitated into diethyl ether several times and the products were obtained after dried in vacuum.

Experimental Setup

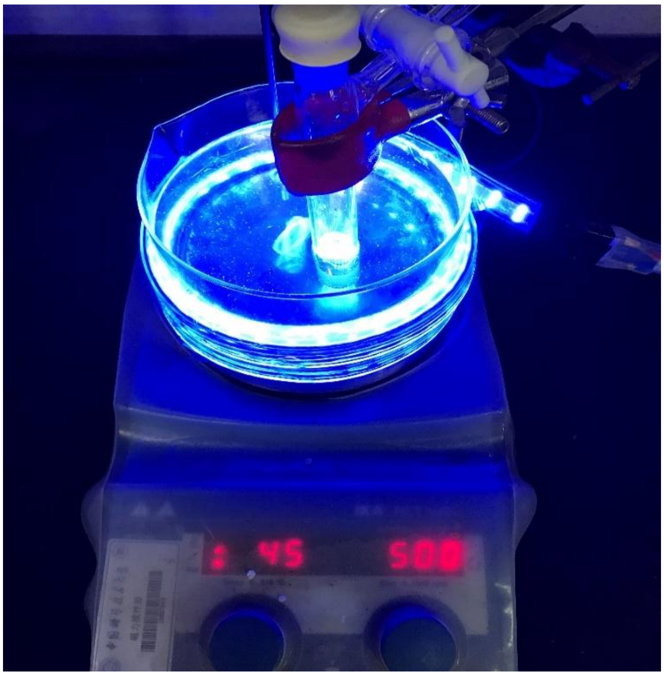

Supplementary Reference

[1]

a) W. C. Fan, L. Wang, S. X. Zheng, *Macromolecules* **2009**, *42*, 327-336; b) P. R. Couchman, F. E. Karasz, *Macromolecules* **1978**, *11*, 117-119.
